# Comparison of proliferation and differentiation of osteoblasts derived from vertebral body and lamina in patients with thoracolumbar kyphosis secondary to Ankylosing Spondylitis

**DOI:** 10.1186/1748-7161-10-S1-P6

**Published:** 2015-01-19

**Authors:** Bangping Qian, Jun Hu, Jing Guo, Mingliang Ji, Xinqiang Wang, Yong Qiu

**Affiliations:** 1 Spine Surgery, the Affiliated Drum Tower Hospital of Nanjing University Medical School, China

## Background

Ankylosing spondylitis (AS) is a chronic inflammatory rheumatic disease. Osteoporosis is a common feature of AS, with a prevalence of 19% to 62%. Previous studies have indicated the increase of osteoclast activity responsible for the development of osteoporosis in AS. However, to our best knowledge, the osteogenic activities of osteoblasts in AS has never been addressed before. Therefore, the objective of this study was to investigate the proliferation and differentiation of osteoblasts derived from the vertebral body and lamina in AS patients and to compare the differences of osteoblast activities between AS and age-matched controls.

## Methods

Fourteen patients with severe AS (13 males and 1 female) underwent pedicle subtraction osteotomy (PSO) were recruited for the present study. Osteoblasts were isolated from trabecular bones harvested from the vertebral body and lamina intraoperatively. Meanwhile, eight men and two women diagnosed as spinal fractures were recruited as the control subjects. Trabecular bones were obtained from the vertebral bodies of patients. The proliferation of osteoblasts derived from controls and AS patients was assessed using the MTT assay, and the differentiation was determined by alkaline phosphatase (ALP) activity using p-nitrophenyl phosphate (p-NPP) as substrate.

## Results

For MTT assay, the optical density (OD) value of the osteoblasts from AS vertebral body was found to be 0.293±0.011; the OD value of the osteoblasts from AS lamina was found to be 0.301±0.015; meanwhile, the OD value of the osteoblasts from controls was found to be 0.298±0.017. There was no significant difference in the proliferation ability among the 3 types of osteoblasts (P = 0.352). For ALP activity assay, the OD values of the osteoblasts derived from AS vertebral body, lamina and controls were 0.286±0.012, 0.285±0.010 and 0.294±0.011, respectively. No difference was found in the differentiation ability among the 3 types of osteoblasts (P = 0.122).

## Conclusion

Osteogenic activities of osteoblasts derived from vertebral body and lamina in AS patients were comparable to that of the osteoblasts from age-matched controls. Our results imply that osteopenia/osteoporosis seen in AS might not be directly attributed to the malfunction of osteoblasts itself.

